# Research on the risk transmission mechanism of international construction projects based on complex network

**DOI:** 10.1371/journal.pone.0285497

**Published:** 2023-08-15

**Authors:** Yanjun Huangfu, Jingrong Xu, Yang Zhang, Dechun Huang, Jiahui Chang

**Affiliations:** 1 Business School, Hohai University, Nanjing, Jiangsu, China; 2 Jiangsu Provincial Collaborative Innovation Center of World Water Valley and Water Ecological Civilization, Nanjing, Jiangsu, China; 3 Business School, Jiangsu Second Normal University, Nanjing, Jiangsu, China; American University of Sharjah, UNITED ARAB EMIRATES

## Abstract

The risk transmission process between international construction projects largely contributes to the dilemma of risk management of international construction projects. Firstly, this paper adopts methods such as literature review and brainstorming to identify the risks in international construction projects from all aspects and all stages. Connections between risks is built by the Delphi method and further construct the international construction project risk network. Combined with “ucinet”, a network visualization analysis tool, overall feature parameters and local feature parameters are presented for analysis as the focus. Starting from this, the risk transmission in complex construction projects is analyzed to identify key risks and transmission relationships and reveal inherent laws of risk transmission. Accordingly, when formulating risk prevention strategies for international engineering projects, it is proposed that measures to curb risk transmission should be effectively adopted from both key risks and their transmission relationships.

## 1. Introduction

International construction projects are complex and highly integrated projects with long cycles and high inputs. They are closely linked to people’s livelihoods and are of great significance to the national economy and social development (Flyvbjerg, 2014 [1]). Participants from different countries and cultures collaborate throughout the entire lifecycle of the construction project(Liu et al, 2021 [2]). However, international construction projects involve many aspects such as the host country’s macro-political and economic situation, international relations, financial markets, import policies, policies and regulations on funds and labor, foreign exchange controls, as well as differences in cultural communication, technical standards, geographical and climatic conditions. Therefore, implementation of the project is relatively difficult, and the risks involved are complex and variable (Behzadi et al. 2018 [3]; Akgul et al., 2017 [4]; Zhu et al., 2022 [5]; Viswanathan, 2020 [6]). Compared to domestic construction projects, international construction projects are larger in scale, have longer durations, and have highly uncertain project objectives, as well as complex interactions with the environment, among other challenges (Ganbat et al., 2020 [7]).

During the implementation of international construction projects, there is usually an interaction relationship between various Risks, rather than isolation, which further complicates the difficulty of Risk Management and Control of international construction projects. For example, during the construction of the Myitsone Dam in Myanmar, due to the prolonged and relentless efforts of Western NGOs to instigate a boycott, the Myanmar government announced a long-term moratorium on the construction of the project on the grounds of opposition from the local population and various sectors of Myanmar society (Zhang et al, 2021 [8]). Risks are interconnected and influence each other, which is the root cause of risk events in international construction projects. From the perspective of the risk evolution process, risk transmission can reduce the risk of international construction project risk sources themselves, but it is also easy to bring about the “domino” effect. Unclear risk transmission mechanisms can easily lead to the dynamic evolution of low-risk events in international construction projects into high-risk major events, bringing serious losses to the whole international construction project. Therefore, how to clarify the interactions between risks in international construction projects so as to analyze the risk transmission mechanism has become an urgent scientific problem to be solved in the current international construction project practice situation.

The risk transmission in international construction projects is essentially the transmission path and process between risk nodes. Complex network analysis has been widely used in risk management (Wang et al, 2019 [9]; Zheng et al,2016 [10]; Dadpour et al, 2019 [11]; Yang et al,2016 [12]), and once the correlation between risk nodes in an international construction project is created, it is a complex network. Therefore, with the help of the complex network theory, this paper intends to reveal the interaction between various risks in international construction projects, identify the core starting and transmission risk elements in the whole life cycle process, and analyze the risk transmission process and mechanism of international construction projects, with a view to providing theoretical innovation and technical reference for international construction project risk management. Through in-depth research, this paper intends to reveal the following questions:

1. What are the risks involved in the different life cycle stages of international construction projects?
2. What is the risk transmission mechanism in international construction projects?

## 2. Literature review

### 2.1 International construction projects in the construction industry

The construction industry places great importance on large construction projects, which serve as a cornerstone of global economic and social development. International construction is a complex form of international cooperation that involves numerous resources such as capital, labor, materials, equipment, and technology. It also involves various international activities such as financing, trade, partnerships, foreign investment, multinational operation, international leasing, and international dispatch. These projects are typically characterized by massive investments, long construction periods, wide-ranging impacts, and the involvement of many stakeholders. They are of strategic importance to the social and economic development of the host country, and include projects such as high-speed railways, highways, photovoltaic power generation, hydropower construction, minerals, ports, and wharves.

Compared with domestic construction projects, international construction projects show their distinctive features when they are in different legal, political, economic, cultural, and social environments (liu et al., 2016 [13]). International construction projects are beneficial to the complementarity of advantages and resources of different countries and to the rapid development of the construction industry and other related businesses. For the host government, it can complete its needs for construction projects by selecting the most suitable international contractor on a global scale, which is conducive to reducing its project costs and absorbing more advanced technology and management concepts and is often more likely to receive high attention from the host government and leaders.

Complexity is one of the most important and basic features of international construction projects. The complexity of international construction projects is determined by the differences and dependencies of many interacting components (Erol et al., 2020 [14]). The combination of the fierce competition in the international market, technical barriers, political factors, cross-border and diverse organizational management, and economic and cultural factors forms the complexity of international construction projects. Specifically, the complexity of international construction projects includes their technical, organizational, and environmental complexity (Gaji´c et al., 2019 [15]; Qazi et al, 2021 [16]). In addition, international construction projects usually involve more stakeholders with complex backgrounds, further increasing the complexity of international construction projects and increasing the resistance to their construction. It is worth noting that during the construction of international construction projects, the internal and external environment is always in a dynamic state of change, and the interests of different stakeholders are also constantly changing. As a result, the organizational structure of international construction projects needs to be constantly adapted at different life cycle stages to respond to changes in internal and external demand. Due to the strategic importance, complexity, dynamic nature and the wide impact of international construction projects, even small changes in the system can generate a variety of risks. Therefore, the successful implementation of international construction projects depends to a great extent on how the risk is managed (Heravi et al.,2018 [17]).

### 2.2 Risks of international construction projects

Project risks are uncertain events or conditions that may arise during the implementation of a project, and will exert a positive or negative impact on the project’s original objectives when occurring (Hoseini et al., 2021 [18]), including schedule planning, budget, quality, scope, safety, and customer satisfaction (Qazi, A et al., 2019 [19]). Risks in international construction include two main categories: systemic and non-systemic risks, of which the systemic one is the risk of loss due to the influence of political, economic, social, and legal environments beyond the control of the enterprise or project decision makers during the implementation of a project (Yan et al., 2021 [20]). Generally speaking, this type of risk is highly contingent and therefore cannot be accurately predicted, while non-systematic risks are those that arise during the implementation of an international construction project and are linked to the project itself (Irem, D et al., 2018 [21]). The systemic risks of international construction projects include political, economic, legal, and society risks throughout all stages of the project. Non-systematic risks include bidding risk, financing risk, and risk of inadequate preparation during the decision preparation stage, as well as cost overrun risk, quality risk, delay risk, technical risk, supply risk, and natural risk during the project implementation stage (Jin et al., 2021 [22]).

However, most of the views are that international construction project risks will have a negative impact on the project (Wang et al., 2016 [23]). As international construction risks continue to arise, scholars have shifted their research perspective to the international construction project risk management. In general, risk management for international construction projects mainly includes risk identification, risk assessment, risk decision, risk monitoring and risk response (Song, 2020 [24]). And the risk identification is the first and most important step in risk management for international construction projects, and it is essential for developing appropriate risk response strategies (Deng et al., 2018 [25]). Scholars have mainly identified risks in international construction projects from the perspective of project lifecycle stages, risk sources, risk effects, the impact of the relationship between risks and the construction projects, or a combination of these. In fact, it is difficult to try to identify all the risks faced by international construction projects. The key of risk management of international construction projects is therefore the identification and management of key risks. Dandage et al. (2018) [26] attempted to assess and rank the main risks in international projects and concluded that political risks, design-related risks, and technical risks were the top three types of risks in international projects.

Risk assessment is a basic method of risk management for international construction projects. Risk assessment for international construction projects includes expert experience and qualitative risk analysis based on subjective judgment (Alamdari et al., 2021 [27]; Qammaz et al., 2021 [28]). Although simple and practical, this method does not take full advantage of information from expert evaluations and fails to take risks and resistance into account, leading to the results of a particular project’s analysis being of little significance to other decision-makers. Therefore, many scholars are very concerned about using theoretical approaches such as the decision-making models to further improve the risk assessment methods, forming the uncertainty analysis model based on the fuzzy set (Wang et al., 2019 [29]; Andrić et al., 2019 [30]). Traditional risk assessments tend to focus on individual risk factors (Guan et al.,2020 [31]), an approach that cannot build a holistic framework for the problem under study and is limited to a simple overlay of risk quantification, which makes it difficult to reflect the interrelationship of various risk variables. Therefore, this study reveals the dynamic changes between risks in different life cycle stages of international construction projects through the complex network analysis, which is conducive to the formulation of risk management strategies for international construction projects in a dynamic environment.

### 2.3 Research on the application of complex network theory

The complex network is an important part of scientific research on complexity. A complex system composed of a set of elements and a set of interactions between these elements can be called a complex network. In fact, complex systems can enable us to understand a variety of real systems. Research on complex networks is inspired by the research and analysis of the real network. The complex network provides a tool to describe natural phenomena, social relations, technologies, and other phenomena of complex systems in the real world, for example, social networks (Zaman S U et al., 2019 [32]), trade networks (Xi et al., 2020 [33]), and financial networks (Qian et al., 2019 [34]) and so on. With the continuous in-depth research by many scholars, complex networks have become a highly active interdisciplinary field integrating computer science, statistics, physics, social science, etc. Through the complex network analysis, Demertzis K et al. (2020) [35] revealed the phenomenon and mechanism of epidemic transmission and promoted research on the anticipation and control of epidemic diseases. Xiao et al. (2019) [36] applied a complex network application model and analyzed the dynamic diffusion of social network information from the perspective of users’ social behaviors and psychological characteristics. Liao et al. (2019) [37] built a relational network of the exchange rate between countries along “the Belt and Road Initiative” and studied the risk contagion structure. Li et al. (2021) [38] introduced the information theory, built the risk spillover network for financial systems, and found that the nonlinear measure is an effective way to develop financial networks and explore risk spillover mechanisms.

In addition, many scholars have adopted the complex network theory to address problems in the field of project management. Based on analyses of industrial project construction, the chain of construction, agent relationships and agent networks, Han.et al. (2021) [39] established a complex network of industrial project construction and construction agents and applied the theory of complex network structural hole to study the non-repetitive relationship between agents in industrial projects. Herrera, RF et al. (2020) [40] used complex network indexes and temporary social diagrams generated within the organization to understand the interactions in construction project design teams. Chen et al. (2022) [41] abstracted the large engineering project (LEP) structure into a multi-layer heterogeneous network consisting of a stakeholder network and a project schedule network and proposed a method to characterize the coupling relationship between the two layers of the heterogeneous network, based on which the correlated scheduling risk analysis model (CSRAM) was improved to determine the transmission mechanism of delayed payment risk in the actual LEP.

The international construction project is a strong national tool to enhance a country’s international competitiveness, and the most powerful “material” used to tell Chinese stories in a more appealing way and shape China’s international image. Meanwhile, international construction projects usually have a span of time and space, a large investment scale, and a far-reaching impact on the host country’s society and economy. While once the risks of international construction projects occur, negative effects of varying degrees can be brought about, and the ensuing governance pain points can affect the social, economic, and environmental development of the host and home countries over a long period of time and space. Therefore, the correlation between risks needs to be analyzed on the basis of the identification of risk elements in international construction projects, and the risk transmission mechanism needs to be clarified through the network nature of the international construction project organization. Therefore, this study combines the complex network theory with the risk transmission in international construction projects to further investigate the nature of nodes and edges in complex networks and their interactions and reveal the risk transmission mechanism in the whole life cycle stages of international construction projects.

## 3. Research method

**Fig 1:**
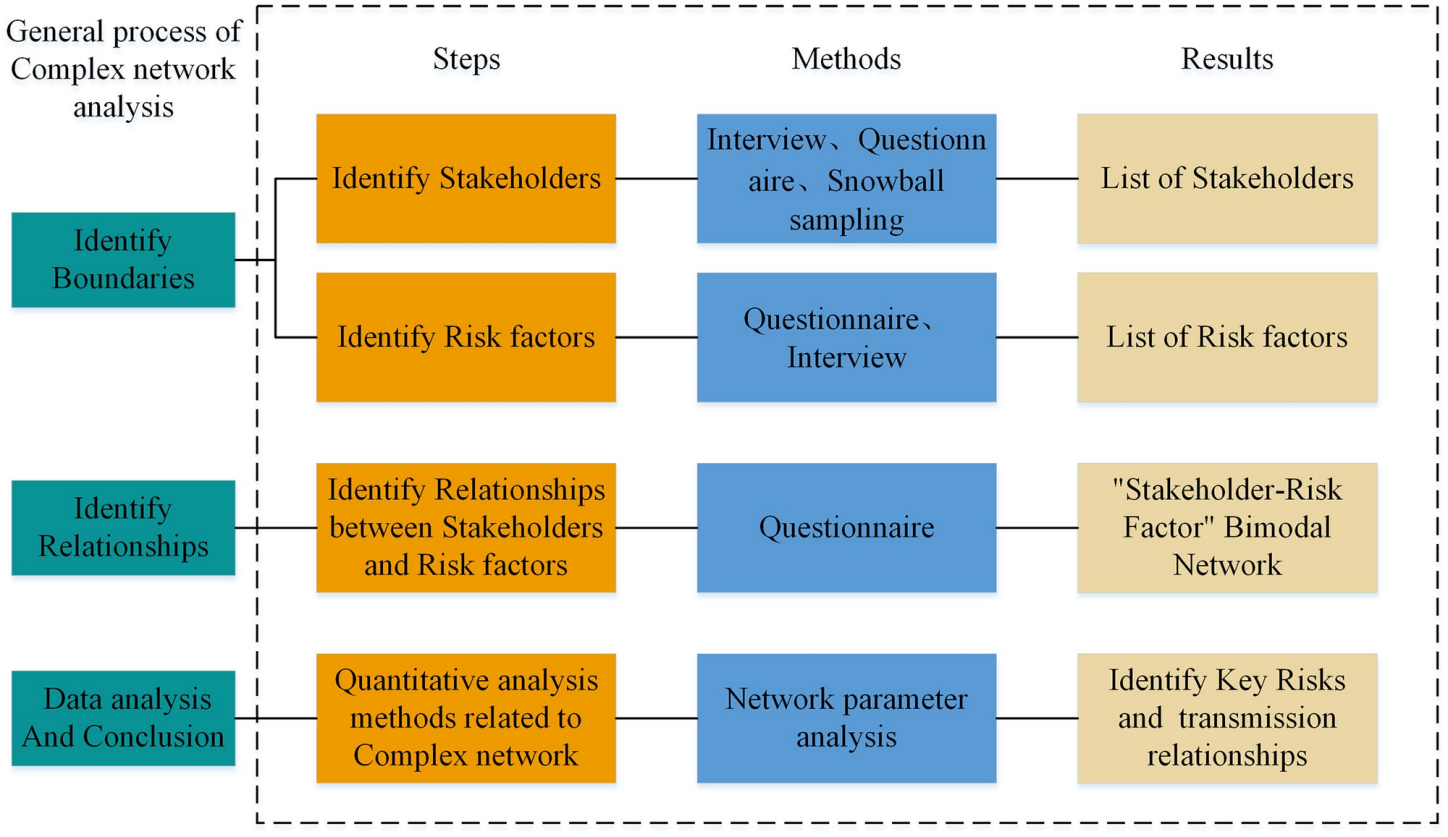
Framework of research methods.

The international construction project is essentially an open, dynamic, and adaptive system where various components influence each other and with a high degree of uncertainty. It is often faced with a more complex risk system than domestic construction projects. Existing studies have attempted to isolate individual risks from the project risk system and identify the categories and risk sources to which each risk belongs but have failed to adequately consider the impact of the interaction between international construction project risks on risk management. International construction project risks are interrelated and influence each other, which is the root cause of the formation of international construction project risks. Due to the dynamic nature of international construction risks, it is difficult to find a precise boundary to distinguish the impact of individual risks. According to the analysis of literature review, complex network analysis can break through the traditional risk transmission relationship analysis of international construction projects. Therefore, this study uses complex networks, combined with the whole life cycle theory to describe the net-like interaction relationship among risks of international construction projects and analyze the risk transmission mechanism of international construction projects(Fig 1. Framework of Research Methods).

### 3.1 Step 1: International construction risk identification

Complex network analysis requires the identification of the nodes and edges in the network. In the risk networks of international construction projects, the nodes refer to the risk elements that occur throughout the project’s whole life cycle. This paper uses a literature analysis method and brainstorming to identify and classify existing key literature on the risk factors of international construction projects. From this, all possible risk factors in the project’s life cycle are summarized, and the frequency of each risk factor is counted and concluded. The fuzzy items are then filtered and removed, and risk items with similar meanings are summarized and organized. Finally, 26 risk factors with a frequency of 3 or more are selected as research objects, resulting in the final list of risk factors presented in Table 1

**Table 1:** List of risk factors for international construction projects.

Risk number	Risk category	Whole life cycle	Risk subject
*R*_1_	Decision risk	Investment and Decision stage	Owner Financial institution Government agency Consulting unit
*R*_2_	Financing risk		
*R*_3_	Political risk		
*R*_4_	Consulting risk		
*R*_5_	Survey risk	Planning and Design stage	Design unit Bidding unit
*R*_6_	Design risk		
*R*_7_	Bidding risk		
*R*_8_	Schedule risk	Construction and Implementation stage	Government agency Owner Contractor Supplier Local residents Supervision unit
*R*_9_	Technical risk		
*R*_10_	Organizational risk		
*R*_11_	Land acquisition and resettlement risk		
*R*_12_	Quality risk		
*R*_13_	Management risk		
*R*_14_	Contract risk		
*R*_15_	Ecological and environment risk		
*R*_16_	Procurement risk		
*R*_17_	Supervision risk		
*R*_18_	Economic risk		
*R*_19_	Society risk		
*R*_20_	Interest expression risk		
*R*_21_	Tax risk		
*R*_22_	Handover risk	Operation and Maintenance stage	Government agency Owner Operating unit Tenement
*R*_23_	Operational risk		
*R*_24_	Market risk		
*R*_25_	Competitive risk		
*R*_26_	Loss risk		

### 3.2 Step 2: Determination of the risk relationship matrix

The relationship in complex networks is expressed in the form of relationship data, and different relationships need to be shown with different relationship data, generally in the form of the matrix. The relationship data of complex networks are composed of three aspects including rows, columns, and relationship data. In the risk network matrix of the international construction projects, the column is defined as the starter of the relationship, i.e., the causal side in the causal-effect relationship; and the row is defined to represent the affected party in the relationship, i.e., the effect side in the causal-effect relationship; “1” means the relationship exists and “0” means the relationship does not exist. As the risk elements cannot be causally linked to themselves, the values on the diagonal of the incidence matrix are all “0”. Representing the above data in a matrix form gives us the risk factor relationship matrix A. Since the two risk elements are not necessarily causally related to each other, the adjacency matrix A is not necessarily symmetric, as is shown in Table 2. In addition, this paper refers to (Boutilier et al., 2017 [42]; Xia *et al*., 2022 [43]) and other research methods, and adopts the questionnaire survey method to determine the relationship between international construction project risks by selecting 25 relevant experts from the field of international construction projects and applying expert evaluation method. For example, if *x* experts believe that *R*_*i*_ is closely related to *R*_*j*_, then *Rij* = *x*_*ij*_.

**Table 2:** International construction project risk relationship matrix.

Risk factor	*R*_*1*_	*R*_*2*_	*R*_*3*_		*R*_*n*_
*R*_*1*_	*0*	*X*_*12*_	*x*_*13*_	……	*X*_*1n*_
*R*_*2*_	*X*_*21*_	*X*_*22*_	*X*_*23*_	……	*X*_*2n*_
*R*_*3*_	*X*_*31*_	*x*_*32*_	*0*	……	*X*_*3n*_
……					
*R*_*n*_	*x*_*n1*_	*X*_*n2*_	*X*_*n3*_	……	*0*

The informed consent of all experts participating in the questionnaire was obtained by telephone prior to the publication of this study.

### 3.3 Step 3: Visualization of the stakeholder-risk factors network

Visualization of the international construction project risk network matrix can visually reveal the relationship between stakeholders and risk factors in the international construction project risk network. At present, the relatively common complex network visualization software includes Ucinet, Pajek, NetMiner, etc. Therefore, this paper uses Ucinet to analyze the risk network in different life cycle stages of international construction projects, where *R*_*i*_ represents the risk element number, and the one-way arrow line indicates there is a causal relationship between the risk elements.

## 4. Results

### 4.1 Network level analysis

**Fig 2:**
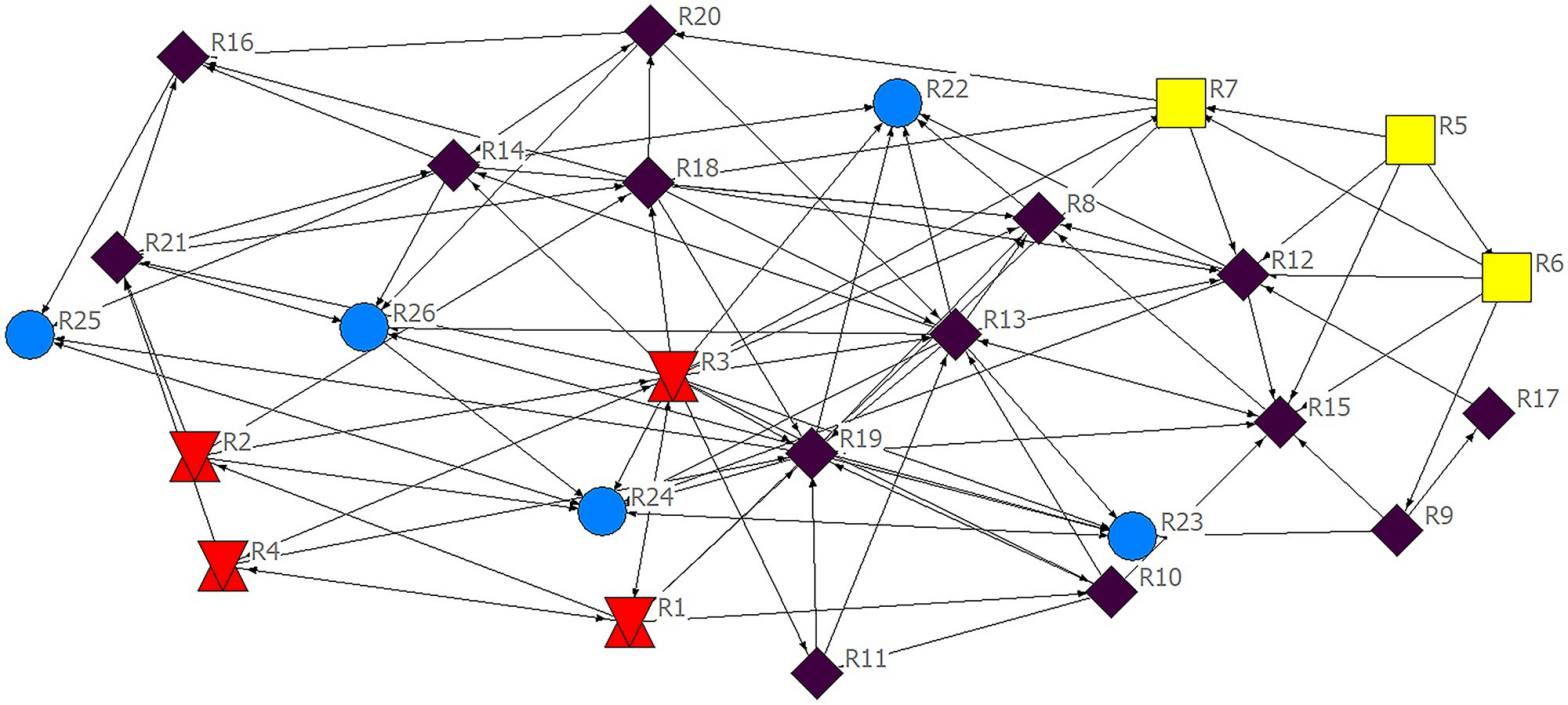
Network distribution of risk factors of international construction projects.

There are 26 nodes and 103 relationships in the risk factor impact network of international construction projects (Fig 2. Network distribution of risk factors of international construction projects). The color and shape of nodes represent risk factors at different life cycle stages. The connection between two nodes reflects the interaction between risk factors. The starting point of the arrow indicates the initiator of the impact relationship, and the ending point of the arrow indicates the receiver of the impact relationship. Risk factors with strong correlation are located in the center of the network, while risk factors with weak correlation are located at the edge of the network.

The average path length can be used to measure the separation distance of nodes in a complex network. The average path length of the risk transmission network model calculated by using “Ucinet” software is 2.096. It shows that the risk network of international construction projects is loosely connected, with an average of about 2 nodes forming a connection between any two risk elements, which has a “small world” feature.

The size of the network clustering coefficient reflects the degree of clustering and connectivity of the network, with bigger network clustering coefficients indicating stronger clustering and connectivity. The average network clustering coefficient calculated by using “Ucinet” is 0.269, while the distribution of the clustering coefficients of the node ranges from 0.167 to 0.417, which shows that the overall degree of clustering and connectivity of the risk network of international construction projects is at a low level. Moreover, the uniform distribution of clustering coefficients from low to intermediate levels across nodes indicates that there are also fewer small associations in networks and less similarity in nature between different risk elements.

The overall network density is the ratio of the actual number of edges between nodes to the total number of possible relationships between nodes and is also a parameter used to measure the connectivity between nodes in the complex network. The higher the network density, the more connectivity paths between the nodes and the stronger the connectivity of the network. The average density of the network calculated by using “Ucinet” is 0.1585, indicating weak connectivity of the project risk network, in line with the analytical structure of the clustering coefficient. In summary, the overall connectivity of the international construction project risk network is weak, the nodes are loosely connected, the network clustering is relatively low, and there is no clustering of associations in the network.

The block model reflects the location of network subgroups and the relationships between them, enabling access to the core risk subgroups of the complex risk network of international construction projects, and thus identifying the key factors and key transmission relationships of the complex risk network of international construction projects. The core subgroups of the complex risk network for international construction projects are obtained by using “Ucinet”. Block 1, block 3, block 4, block 5, and block 6 are located in the transmission position (having both receiving and sending relationships); block 2 and block 8 are located in the sending position (only having sending relationships); and block 7 is located in the receiving position (only having receiving relationships). The blocks at the core of the block model theory should have both a receiving and a sending relationship; therefore, block 1, block 3, block 4, block 5 and block 6 in the transmission position have the prerequisites to be at the core of the block. According to further analysis, block 3 mainly assumes the role of receiving which influences the relationship, and there is only one sending relationship being passed to block 7, which only takes on a receiving role and does not play a sending role and causes the relationship not to be transmitted. And therefore, it can be judged that block 3 is not in a core position. Block 1, block 4, block 5, and block 6 are therefore at the core position of the risk network and are the core blocks of the whole life cycle risk network for international construction projects.

### 4.2 Analysis of network local parameters

In complex networks, local parameters mainly include betweenness centrality analysis, intermediary analysis, and closeness centrality. Among them, the betweenness centrality analysis and intermediary analysis can judge the key risks in the complex network of international construction projects. While the betweenness centrality analysis of the line can identify the relationship between key risks. This study describes the individual network characteristics of the whole life cycle risks of international construction projects through these two parameters.

**(1) Intermediary analysis**

In the social network analysis, intermediaries are generally classified according to Gould and Fernandez’s theory, which includes five roles: coordinator, gatekeeper, agent, advisor, and liaison. Suppose that in a social network, node *A* influences node *C* through node *B*. When three nodes are in different groups, node *B* will play a different role. Node *B* assumes the coordinator role when nodes *A*, *B*, and *C* are in the same group; and node *B* takes on the gatekeeper role when node *A* is in one group and nodes *B* and *C* are in another; when nodes *A* and *B* are in one group and node *C* is in another, node *B* takes on the role of the agent; when nodes *A* and *C* are in one group and node B is in another, node *B* takes on the role of advisor; when nodes *A*, *B*, and *C* are in separate groups, node *B* takes on the role of liaison. The intermediary roles in the risk network for international construction projects are derived from the “Ucinet” analysis, which can be seen in Table 3. The top 1/3 of the risks are usually selected as core risks. Therefore, it can be seen that *R*_3_ (Political risk political risk), *R*_19_ (social risk), *R*_13_ (management risk), *R*_14_ (contract risk), *R*_15_ (ecological and environmental risk), *R*_24_ (market risk), and *R*_21_ (financial and tax risk) are the core risks of the whole life cycle risk network of international construction projects, and these 7 categories of risks together bear 55% of the intermediaries.

**Table 3:** Intermediary analysis results of whole life cycle.

Risk factor	Intermediary Analysis					Total
	Coordinate	Gatekeeper	Represent	Consultant	Liaison	
*R*_*3*_	101	14	0	0	0	115
*R*_*19*_	75	13	23	1	0	112
*R*_*13*_	89	7	0	0	0	96
*R*_*14*_	83	1	0	0	0	84
*R*_*15*_	29	35	12	5	0	81
*R*_*24*_	67	12	0	0	0	79
*R*_*21*_	78	1	0	0	0	79
*R*_*18*_	61	17	0	0	0	78
*R*_*8*_	68	9	0	0	0	77
*R*_*12*_	59	10	0	0	0	69
*R*_*16*_	0	7	27	16	0	50
*R*_*6*_	11	32	0	0	0	43
*R*_*5*_	23	19	4	0	0	46
*R*_*10*_	20	11	1	0	0	32
*R*_*20*_	1	3	20	2	0	26
*R*_*7*_	15	6	0	0	0	21
*R*_*23*_	5	14	0	0	0	19
*R*_*26*_	16	0	0	0	0	16
*R*_*9*_	11	0	2	0	0	13
*R*_*25*_	0	12	0	0	0	12
*R*_*1*_	10	0	0	0	0	10
*R*_*2*_	1	4	0	3	0	8
*R*_*17*_	3	3	0	0	0	6
*R*_*4*_	2	0	2	0	0	4
*R*_*11*_	0	0	0	0	0	0
*R*_*22*_	0	0	0	0	0	0

**(2) Betweenness centrality analysis of the node**

The betweenness centrality of the node refers to the probability of the node falling between other nodes, and the probability indicates the degree of the node playing a bridging role in the network. The higher the betweenness centrality of the node, the greater the probability, and the greater the bridging role, indicating that the node is at the core position of a complex network. In the whole life cycle risk network of international engineering projects, if a node is on more than one transmission path and controlling this node can help control multiple paths so as to curb the risk transmission, then the risk will be considered to be at the core position. The betweenness centrality of nodes of the whole life cycle risk network of international construction projects is calculated by “Ucinet”, which can be seen in Table 4. Referring to the above method, the top 7 risks in terms of betweenness centrality among 26 risks were selected as the core individual risks, therefore, *R*_3_ (political risk), *R*_19_ (social risk), *R*_15_ (ecological and environmental risk), *R*_14_ (contract risk), *R*_13_ (management risk), *R*_24_ (market risk), and *R*_18_ (economic risk) are the core risks of the whole life cycle risk network of China’s overseas water conservancy and hydropower projects. And there is one difference between these 7 core risks and the results of the intermediary analysis.

**Table 4:** Betweenness centrality analysis results of the node.

Rank	Node	Betweenness Centrality of the Node	Rank	Node	Betweenness Centrality of the Node
1	*R*_3_	27.817	14	*R*_26_	4.7
2	*R*_19_	21.908	15	*R*_9_	3.583
3	*R*_15_	21.753	16	*R*_25_	3.5
4	*R*_14_	18.825	17	*R*_6_	2.25
5	*R*_13_	18.611	18	*R*_10_	1.9
6	*R*_24_	15.317	19	*R*_1_	1.7
7	*R*_18_	14.369	20	*R*_2_	1.361
8	*R*_21_	12.928	21	*R*_17_	1.25
9	*R*_16_	11.22	22	*R*_7_	0.833
10	*R*_12_	8.306	23	*R*_4_	0.569
11	*R*_20_	7.905	24	*R*_5_	0
12	*R*_8_	7.903	25	*R*_11_	0
13	*R*_23_	7.392	26	*R*_22_	0

**(3) Betweenness centrality analysis of the line**

The betweenness centrality of the line refers to the probability of the line falling between other lines, and the probability indicates how much control the relationship line has over the whole network. The higher the betweenness centrality of the line, the greater the probability and the stronger the controlling role, indicating that the relationship line is at the core position of the social network. In the whole life cycle risk network of China’s overseas water conservancy and hydropower projects, if the betweenness centrality of a relationship line is higher, the more the relationship line transmission should be controlled to curb the risk spreading. The betweenness centrality of lines of the whole life cycle risk network of international construction projects is calculated by using “Ucinet”. Due to the large number of risk nodes, the complexity of the relationship lines, and the length of the calculated results, they are not shown here and can be asked from the author if any reader needs them.

### 4.3 Identification of key risk factors

By combining the previous analyses of overall network characteristics and individual network characteristics, key risk factors for the whole life cycle of international construction projects can be identified as follows:

Firstly, the result of the intermediary analysis describing the individual network characteristics is that the top 7 risks in the total number of intermediaries among 26 risks are the core individual risks. *R*_3_ (political risk), *R*_19_ (social risk), *R*_13_ (management risk), *R*_14_ (contract risk), *R*_15_ (ecological and environmental risk), *R*_24_ (market risk), and *R*_21_ (financial and tax risk) together bear 55% of the intermediaries, representing the core risks of the whole life cycle risk network of international construction projects. The result of the betweenness centrality analysis of the nodes shows that the top 7 risks in the betweenness centrality among 26 risks are *R*_3_ (political risk), *R*_19_ (social risk), *R*_15_ (ecological and environmental risk), *R*_14_ (contract risk), *R*_13_ (management risk), *R*_24_ (market risk), and *R*_18_ (economic risk), indicating that these 7 types of risks have a greater probability of being in between other nodes and play a greater bridging role. And they are the core risks of the whole life cycle risk network of China’s overseas water conservancy and hydropower projects.

Therefore, according to the individual network characteristics, the core risks of the whole life cycle risk network of China’s overseas water conservancy and hydropower projects include *R*_3_ (political risk), *R*_19_ (social risk), *R*_13_ (management risk), *R*_14_ (contract risk), *R*_15_ (ecological and environmental risk), *R*_24_ (market risk), *R*_21_ (financial and tax risk), and *R*_18_ (economic risk).

Secondly, the overall network characteristics show that block 1 (*R*_15_, *R*_3_, *R*_19_), block 4 (*R*_12_, *R*_18_, *R*_24_, *R*_21_), block 5 (*R*_6_, *R*_16_, *R*_5_), and block 6 (*R*_8_, *R*_13_, *R*_14_) are in the core position of the network risks and are core blocks of the whole life cycle risk network of international construction projects. Among the core risk factors, *R*_3_ (political risk), *R*_19_ (social risk), and *R*_15_ (ecological and environmental risk) are located in core block 1; *R*_24_ (market risk), *R*_21_ (financial and tax risk), and *R*_18_ (economic risk) are located in core block 4; and *R*_13_ (management risk) and *R*_14_ (contract risk) are located in core block 6.

In summary, *R*_3_ (political risk), *R*_19_ (social risk), *R*_13_ (management risk), *R*_14_ (contract risk), *R*_15_ (ecological and environmental risk), *R*_24_ (market risk), *R*_21_ (financial and tax risk), and *R*_18_ (economic risk) are the key risk factors of the whole life cycle risk network of international construction projects.

### 4.4 Identification of key risk relationships

8 categories of the key risk factors for the whole life cycle risk network of international construction projects are obtained through the identification of key risk factors above. But this only shows that these 8 categories of risks should be paid high attention to and does not reveal how the key risks of the whole life cycle risk of international construction projects are transmitted. In addition to the key factors of risk network nodes, the relationships of the overall risk network (the connections between nodes) also play an important role in risk transmission. The calculation of the betweenness centrality of the lines in a risk network can assess objectively the extent to which a relationship is located in other relationships and identify the relationships that are highly communicative in the network so as to improve the objectivity and comprehensiveness of the identification of risk network characteristics. This can further improve the efficiency of risk management and block the transmission of some risks throughout the network powerfully.

Through the analysis of the calculated results of the betweenness centrality of the lines of the whole life cycle risk network of international construction projects, it can be found that there are 103 lines with betweenness centrality larger than 0. Due to the limited space, only the top 10 relationships are taken and included in Table 5 below. As is shown in Table 5, *R*_3_→*R*_19_, *R*_3_→*R*_22_, *R*_3_→*R*_2_, *R*_19_→*R*_15_, *R*_19_→*R*_24_, *R*_13_→*R*_14_, *R*_13_→*R*_19_, *R*_21_→*R*_18_, *R*_3_→*R*_1_, and *R*_13_→*R*_22_ are the 10 relationships with the greatest betweenness centrality of the lines, i.e., they are located with a high probability in other relationship lines and with 5strong control over the overall risk network transmission, and they are the key relationships for the whole life cycle risk transmission of international construction projects. Besides, the nodes corresponding to the relationship lines are closely related to the key risk factors *R*_3_ (political risk), *R*_19_ (social risk), *R*_13_ (management risk), *R*_14_ (contract risk), *R*_15_ (ecological and environmental risk), *R*_24_ (market risk), *R*_21_ (financial and tax risk) and *R*_18_ (economic risk) identified previously. Namely, if the above key risk factors can be effectively managed, their closely related key relationships may not be considered separately.

**Table 5:** Betweenness centrality of the key relationships.

Rank	Relationship	Betweenness Centrality of the Relationship	Rank	Relationship	Betweenness Centrality of the Relationship
1	*R*_3_→*R*_19_	166.408	6	*R*_13_→*R*_14_	61.2
2	*R*_3_→*R*_22_	159.455	7	*R*_13_→*R*_19_	61.2
3	*R*_3_→*R*_2_	137.505	8	*R*_21_→*R*_18_	60.061
4	*R*_19_→*R*_15_	69.655	9	*R*_3_→*R*_1_	59.133
5	*R*_19_→*R*_24_	61.322	10	*R*_13_→*R*_22_	56.474

## 5. Conclusions

This study builds a risk-related network model for the whole life cycle of international construction projects from the perspective of the network and accurately captures the key risks throughout the construction process of international construction projects through methods such as parametric analysis and visual model. After a combination of the above results, it can be found that governmental institutions, owners, and contractors are the major sources of risks in international construction projects in terms of the subject of risk generation. In terms of the stages of risk generation, the key risks in the international construction project risk network are mainly found in the construction and implementation stage and the operation and maintenance stage throughout the project life cycle. These two stages are the most critical stages in the project life cycle, with the longest time span, the largest number of participants and the strongest interactions among different parties. Therefore, the number of risk factors increases exponentially during these stages and interactions between various nodes enhance obviously, with the emergence of risk factors. Through the analyses above, it can be found that the management risk, contract risk, market risk, and financial and tax risk are all important risks emerging in the construction stage, and there is an interaction between these network nodes and many other neighboring risk nodes. Therefore, it plays a significant role in risk transmission. Besides, social risk in the investment and decision stage investment decision stage is also important transmission node that cannot be neglected, indicating that the risks in other stages of the whole life cycle of international construction projects will lead to the occurrence of this risk, and it will also react to other risks. And if such important node is removed, the connectivity and tightness of the whole risk network will be greatly reduced. Therefore, it is an important part of the risk control work of international construction projects to find ways and means to prevent risks from the key nodes of the risk network.

There are certain shortcomings in the research of this paper: this paper uses Delphi method to determine the correlation between risk factors in the whole life cycle of international engineering projects, but Delphi method may lead to unreliability of the research results due to the defects such as incomplete expertise of experts, and we will further optimize the research method in the subsequent research to compensate for the defects of this paper.

## Supporting information

S1 File (XLSX)
